# Fingerprinting of Nitroaromatic Explosives Realized by Aphen-functionalized Titanium Dioxide

**DOI:** 10.3390/s19102407

**Published:** 2019-05-27

**Authors:** Guanshun Xie, Bingxin Liu

**Affiliations:** Qinghai Provincial Key Laboratory of New Light Alloys, Qinghai Provincial Engineering Research Center of High Performance Light Metal Alloys and Forming, Qinghai University, Xining 810016, China; guanshunxie@126.com

**Keywords:** Aphen, TiO_2_, sensor array, nitroaromatic explosives

## Abstract

Developing sensing materials for military explosives and improvised explosive precursors is of great significance to maintaining homeland security. 5-Nitro-1,10-phenanthroline (Aphen)-modified TiO_2_ nanospheres are prepared though coordination interactions, which broaden the absorption band edge of TiO_2_ and shift it to the visible region. A sensor array based on an individual TiO_2_/Aphen sensor is constructed by regulating the excitation wavelength (365 nm, 450 nm, 550 nm). TiO_2_/Aphen shows significant response to nitroaromatic explosives since the Aphen capped on the surface of TiO_2_ can chemically recognize and absorb nitroaromatic explosives by the formation of the corresponding Meisenheimer complex. The photocatalytic mechanism is proved to be the primary sensing mechanism after anchoring nitroaromatic explosives to TiO_2_. The fingerprint patterns obtained by combining kinetics and thermodynamics validated that the single TiO_2_/Aphen sensor can identify at least six nitroaromatic explosives and improvised explosives within 8 s and the biggest response reaches 80%. Furthermore, the TiO_2_/Aphen may allow the contactless detection of various explosives, which is of great significance to maintaining homeland security.

## 1. Introduction

Common explosives could be classified into military explosives (e.g., nitroaromatic explosives like 2,4,6-trinitrotoleune (TNT), 2,4-dinitrotoleune (DNT), 2,4,6-trinitrophenol (PA), etc.), and improvised explosives such as ammonium nitrate (NH_4_NO_3_), sulfur (S) and triacetone peroxide (TATP), etc. Developing sensors against military explosives or improvised explosive precursors has important significance for maintaining global security. At present, there are many methods for detecting these explosives, of which, the most traditional method is the use of sniffer dogs. However, sniffer dogs have lower working efficiency than sensors since they need a rest for half an hour after working for 20 min. In addition, existing explosive detection methods that are more mature include X-ray inspection technology, neutron analysis [1,2], fluorescence detection [3,4], electrochemical detection [5], mass spectrometry [6], ion migration method [7], and chromatography [8], etc. However, neutron analysis and X-ray detection instrument are cumbersome, expensive, and radiation hazard. Chromatographic methods are generally only suitable for laboratory sampling and testing and cannot meet the purpose of rapid detection on site. In addition, these methods are mostly contact-based detection methods. Furthermore, there are relatively few studies on detection of improvised explosives, limiting their application on the actual detection of explosives. Therefore, there is an urgent need for rapid vapor phase detection of conventional and improvised explosives with a small, low energy consumption, high sensitivity, rapid response, and contactless photoelectronic sensor.

Construction of a sensory array that is similar to an artificial olfactory system and utilizing the cross-sensitivity of different sensors towards complex component gases, and combining data analysis methods can achieve the identification of different explosive vapors [9,10]. However, the stability of the sensor array [11,12] is dependent on the synergistic effects of the different sensor units, and failure of a single unit can affect the performance of the sensor array. To this end, our previous work constructed a sensor array using a single sensor unit by illumination of different excitation wavelengths [13].

Titanium dioxide (TiO_2_) is a photoelectronic sensor candidate due to its chemical stability, environment friendliness and excellent optical response [14,15,16]. However, pure TiO_2_ can only absorb ultraviolet light due to its relatively wide band gap (3.2 eV) [17]. Moreover, the high recombination rate of photoinduced electron-hole pairs hinders the practical application of TiO_2_ in the field of sensors. 5-Amino-1,10-phenanthroline (Aphen) [18,19,20] is an excellent electron acceptor that can chelate with most metal ions to form stable complexes via coordination interactions. In this study, Aphen is employed as a functional ligand to modify TiO_2_ nanocrystals to promote the photoinduced charge carrier separation efficiency of the nanocrystals. Moreover, it is well known that the electron-rich amino group of Aphen can link with electron-deficient aromatic rings of nitroaromatic explosives to formation of Meisenheimer complexes. Thus, the Aphen-modified TiO_2_ nanocrystal is promising for discrimination of nitroaromatic explosives.

## 2. Materials and Methods

### 2.1. Experiment Reagents and Equipment

Tetrabutyltitanate (99%, Aladdin, Shanghai, China), concentrated sulfuric acid (AR, Wokai, Shanghai, China), hydrofluoric acid (AR, Aladdin), anhydrous ethanol (99.5%, Hushi, Shanghai, China), phenanthroline monohydrate (99%, Aladdin), and deionized water were used.

Field emission scanning electron microscopy (SEM, JSM-6610LV, JEOL, Ltd, Tokyo, Japan) and transmission electron microscopy (TEM, JEM-2100F microscope, JEOL Ltd, Tokyo, Japan) experiments were performed to observe microstructure. X-ray diffraction (XRD) patterns were measured using a D8 Advance X-ray diffractometer (Bruker Ltd, Bremen, Germany) at a scanning rate of 6° min^−1^ with 2θ ranging from 20° to 80°, using CuKα radiation (λ = 1.5418 Å). FTIR spectra were recorded on a Magna 560 FT-IR spectrometer (Nicolet Ltd, Green Bay, WI, USA). UV/Vis absorption spectra were recorded on a UV-2550 UV/Vis spectrometer (Shimadzu, Japan) in the range 200–800 nm. An X-ray photoelectron spectrometer (XPS, PHI5000 ESCA, Perkin Elmer, Waltham, MA, USA) equipped with an Al Kα source (1486.6 eV photons) was used to characterize the modifying of Aphen on TiO_2_. A Keithley 4200A-SCS parameter analyzer (Tektronix Ltd, Beaverton, OR, USA) was used to record the photocurrent of sensor chip in different explosive vapors.

### 2.2. Preparation of Aphen-functionalized Titanium Dioxide Nanosphere

#### 2.2.1. Preparation of 5-amino-1,10-phenanthroline (Aphen)

Phenanthroline monohydrate (50.4 mmol) was mixed with concentrated sulfuric acid (7.5 mL), and heated before a mixture of concentrated sulfuric acid and concentrated nitric acid (1:1, 30 mL) was slowly added drop by drop. The mixture was refluxed for 3 h under 160 °C to obtain 5-nitro-1,10-phenanthroline. The synthesized 5-nitro-1,10-phenanthroline is rapidly added to an ethanol solution containing 5% Pd/C catalyst (0.01 g) and hydrazine hydrate (45 mmol) at 70 °C and refluxed for 2 h. The solution was cooled, filtered, and crystalized for subsequent experiments to obtain 5-amino-1,10-phenanthroline [21].

#### 2.2.2. Preparation of TiO_2_ Nanocrastals

Tetrabutyltitanate (25 mL) and concentrated sulfuric acid (3 mL) were added to a reaction kettle and then hydrofluoric acid (0.17 mol) was added. The mixture was allowed to react at 180 °C for 24 h. After the reaction kettle has cooled to room temperature, then the product was washed by deionized water and ethanol repeatedly and dried in a vacuum drying oven.

#### 2.2.3. Preparation of Aphen-functionalized TiO_2_ Nanocrystals (TiO_2_/Aphen)

A mass ratio of 1:50, 1:100, 1:200 of Aphen and TiO_2_ nanocrystals was placed in a 1 mL round-bottomed flask and vigorously shaken for 10 h at 60 °C. Then the nanocrystals were repeatedly washed with deionized water and ethanol until the wash solution does not emit light under an UV lamp. The product was vacuum-dried.

### 2.3. Preparation of Sensor Chips

Electrochemical deposition was used to deposit a gold interdigitated electrode with a width and interval of 3 mm at the ceramic substrate (10 mm × 9.5 mm). The gold thickness of the surface layer of the electrode was 0.5 mm. The TiO_2_/Aphen ethanol dispersion was spin coated on the interdigitated electrode and air-dried. The coating was aged for 12 h at a voltage of 4 V before use.

### 2.4. Gas Sensing Properties Testing

2,4,6-Trinitrotoluene (TNT), 2,4-dinitrotoluene (DNT), picric acid (PA), nitrate (NH_4_NO_3_), sulfur (S) and triacetone peroxide (TATP, diluted to 600 ppb by air) vapor were evaporated in advance to saturate. The photoelectric and gas sensitivity response performance of the TiO_2_/Aphen sensor chip to these six conventional and improvised explosives were recorded by the Keithley 4200A-SCS parameter analyzer under 365 nm, 450 nm, 550 nm monochromatic light. After the gas sensor chip was stabilized for half an hour in air under a voltage of 4 V, it was placed into a test bottle that contained saturated explosive vapor. The chip was removed after the current was stable. This process was carried out three times.

## 3. Results and Discussion

### 3.1. TiO_2_@Aphen Morphological and Structural Characterization

**Scheme 1:**
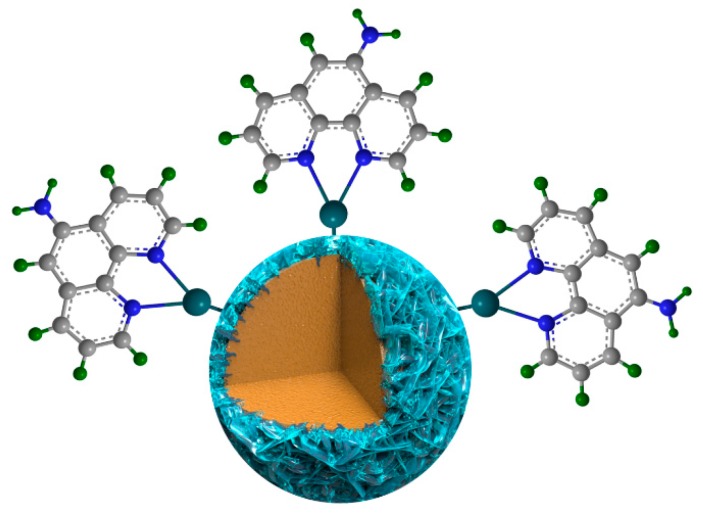
Schematic illustration for the Aphen-functionalized TiO_2_ nanospheres through coordination interactions between Aphen and Ti ions dangling on the surface of TiO_2_.

The TiO_2_ prepared by hydrothermal method was first modified by F ions and then doped with Aphen since the Aphen has high affinity to Ti ions dangling on the surface of TiO_2_ as depicted in Scheme 1. The TiO_2_ morphology was adjusted by using F ions. The tetrabutyltitanate underwent hydrolysis under acidic conditions to grow different TiO_2_ morphologies. As shown in Figure S1, when F ions were not present, the morphology of TiO_2_ appeared as aggregated nanosheet structures. When 0.03 mol of HF was added, the schistose titanium dioxide began to appear flocculent. When 0.06 mol of HF was added, the microstructure of titanium dioxide is changed to a cluster of many nanosheets. When 0.12 mol of HF was added, the morphology of TiO_2_ appeared as cubes with 1 µm sides.

**Figure 1:**
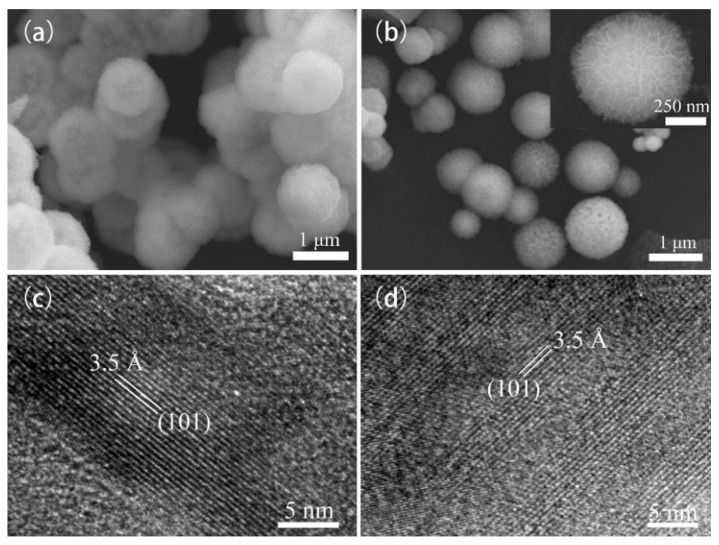
SEM images (**a**,**b**) and HRTEM (**c** and **d**) images of TiO_2_ nanospheres before (**a**,**c**) and after (**b**,**d**) Aphen modifying. Inset: SEM images of single Aphen modified TiO_2_ nanocrystals.

Figure 1a shows that the litchi-like TiO_2_ nanospheres with streaks on the surface was regulated by F ions with a diameter ranging from 12 to 18 μm when 0.09 mol of HF was added, and these nanospheres aggregate each other. Then Aphen chelates with TiO_2_ nanospheres since the Aphen has high affinity to Ti ions overhung on the surface of TiO_2_. Obviously, Aphen modified TiO_2_ has favorable dispersibility as depicted in Figure 1b, which is roughly attributed to the steric effect of Aphen modified on the surface of TiO_2_ nanospheres. The enlarged SEM image (Figure 1b inset) clearly shows that the surface of litchi TiO_2_ nanosphere is a disordered TiO_2_ nanosheet growing vertically in the sphere. Figure 1c,d are the HRTEM diagrame of the edge of a nanosheet on a spherical shell of TiO_2_ and TiO_2_/Aphen. According to the measurement, both the lattice fringe spacings are 0.35 nm, corresponding to (101) planes of TiO_2_ nanocrystals. It is worth mentioning that the crystalline structure of the TiO_2_ remained intact after Aphen functionalization.

**Figure 2:**
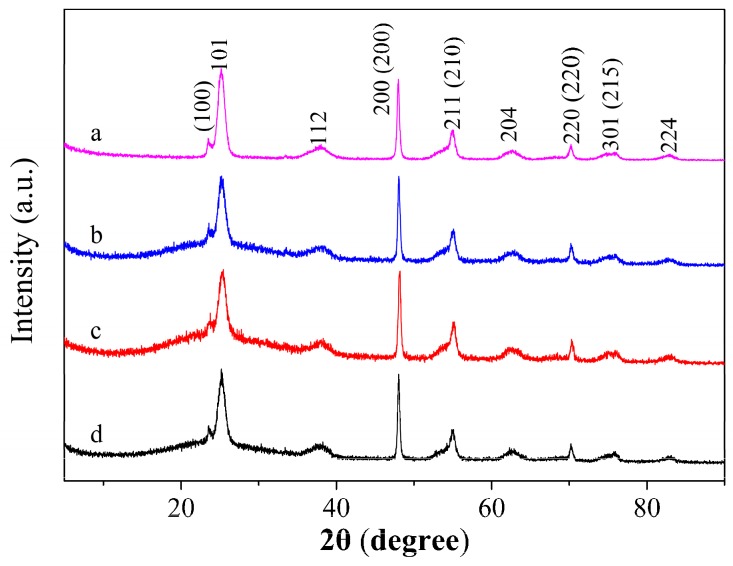
XRD pattern of TiO_2_ nanospheres before (**a**) and after modifying Aphen with Aphen/ TiO_2_ ratio of 1:50 (**b**); 1:100 (**c**) and 1:200 (**d**).

The crystallinity of the TiO_2_ nanospheres and TiO_2_/Aphen were confirmed by XRD. As shown in Figure 2, the XRD pattern (Line a) of TiO_2_ nanospheres shows sharp diffraction peaks at 25.0°, 38.2°, 47.9°, 55.1°, 62.8°, 70.2°, 75.6°, 82.9°, which are indicative of the (101), (112), (200), (211), (204), (220), (301), (224) characteristic planes of the anatase TiO_2_ crystal [22,23,24].

Moreover, characteristic planes of TiOF_2_ are also obtained at 23.6°, 48.1°, 54.2°, 70.5°, 75.2°, which are indicative planes of the (100), (200), (210), (220) and (215) [25,26,27] (PDF-2 Release 2015 RDB). It could be concluded that a small part of the precursor transformed into TiOF_2_ crystals after F ion modifcation and these TiOF_2_ dangling on the surface of TiO_2_ nanospheres, which supplies an adequate number of Ti ions to anchor Aphen through coordination interactions (Scheme 1). However, there are only the characteristic diffraction peaks of TiO_2_@TiOF_2_ phase when TiO_2_ was modified by Aphen at different ratios (Line b to d: 1:50, 1:100, 1:200), which is attributed to the very low content of the Aphen on the surface of TiO_2_. This result also indicates that the modification of Aphen does not change the crystal structure of TiO_2_@TiOF_2_.

**Figure 3:**
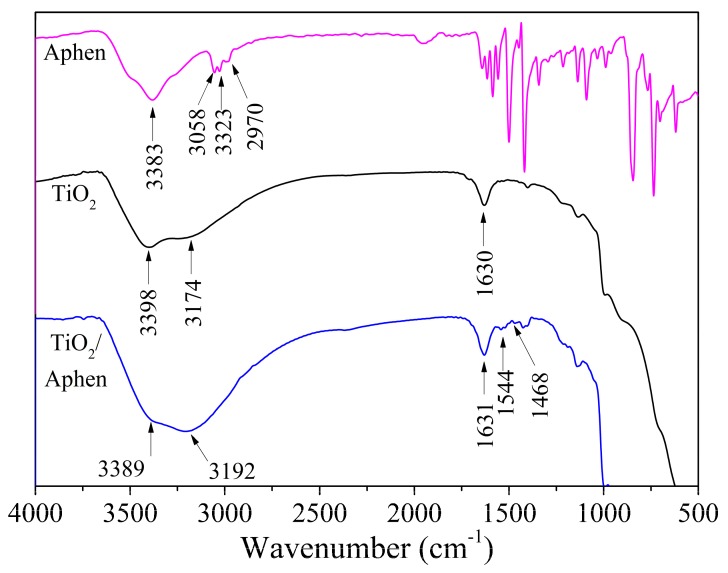
FTIR spectra of Aphen, TiO_2_, and TiO_2_/Aphen (1:200).

Figure 3 shows the Fourier Transform infrared spectra (FTIR) of Aphen, TiO_2_, and TiO_2_/Aphen. The characteristic bands observed in as-synthesized Aphen at 3383 cm^−1^ are attributed to the stretching vibration peak of N=H, and 2970 cm^−1^, 3323 cm^−1^ and 3058 cm^−1^ bands are attributed to the stretching vibration peak of the aromatic ring skeleton. The broad characteristic band of TiO_2_ is observed at about 500 cm^−1^. Peaks at 3398 cm^−1^ and 3174 cm^−1^ are the stretching vibration peaks of -OH moieties on the surface of TiO_2_, which is attributed to the presence of water adsorbed on the surface of TiO_2_ and dangling hydroxyls on the surface of TiO_2_ [28,29,30,31]. When Aphen is anchored to the surface of TiO_2_, the obvious stretching vibration peaks of C=N on Aphen can be observed at 1468 cm^−1^ and 1544 cm^−1^. The peak of 3192 cm^−1^ is caused by the overlapping of the -OH vibration peak in TiO_2_ and the C=C vibration peak of the benzene ring in Aphen. These results indicate that the surface of TiO_2_ is is successfully modified with Aphen.

**Figure 4:**
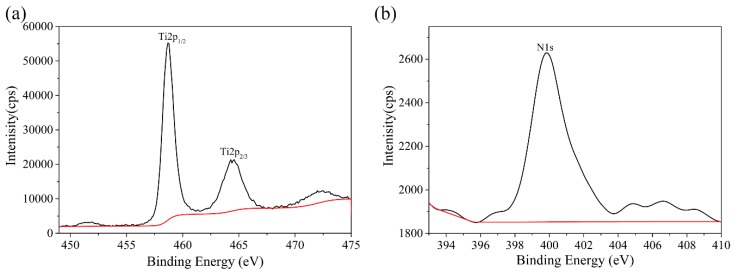
XPS spectra of Ti (**a**) and N (**b**) of TiO_2_/Aphen.

The Aphen-modified TiO_2_ was also analyzed by X-ray photoelectron spectroscopy (XPS). As shown in Figure 4, the binding energy (BE) of Ti 2p from Ti 2p_1/2_ and Ti 2p_2/3_ can be detected at 458.72 eV and 464.45 eV. The XPS spectra of N 1s in Aphen shows the BE at 339.85 eV. This is further proof for the adsorption of Aphen onto surface of the TiO_2_. Moreover, the XPS measurements were performed to reveal the atomic content. Based on the peak areas of Ti and N, it can be concluded that approximately 3.5% of the N on the surface of the TiO_2_.

**Figure 5:**
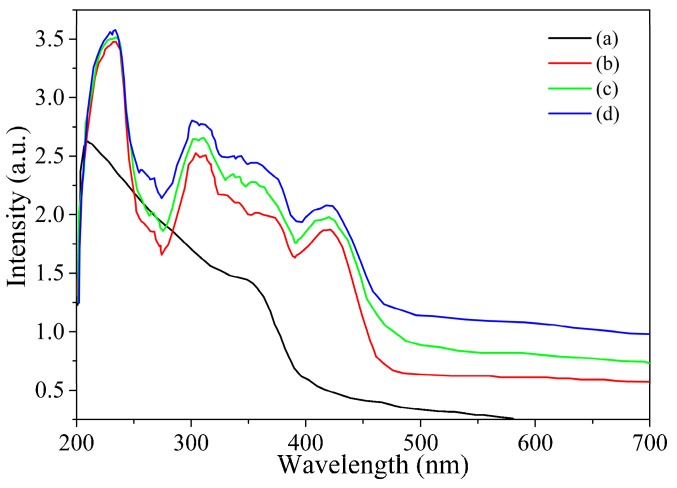
UV-vis absorption of TiO_2_ nanospheres before (**a**) and after modifying Aphen with Aphen/ TiO_2_ ratio of 1:50 (**b**); 1:100 (**c**) and 1:200 (**d**).

UV-visible absorption spectra of TiO_2_/Aphen were recorded to prove the effect of Aphen on the photoelectric properties of TiO_2_. As shown in Figure 5, the anatase TiO_2_ naospheres shows a broad absorption wavelength in the ultraviolet region from 200 nm to 410 nm. Interestingly, the absorption of Aphen-modified TiO_2_ broaden the absorption band edge into the visible region. Therefore, the results show that the ultraviolet absorption wavelength of Aphen-functionalized TiO_2_ is greatly extended, which can avoid the risk of explosive explosions caused by high-energy electron beam as much as possible when detecting explosives by the photoelectric effect [32].

### 3.2. Gas Sensitivity Performance of TiO_2_@Aphen

**Figure 6:**
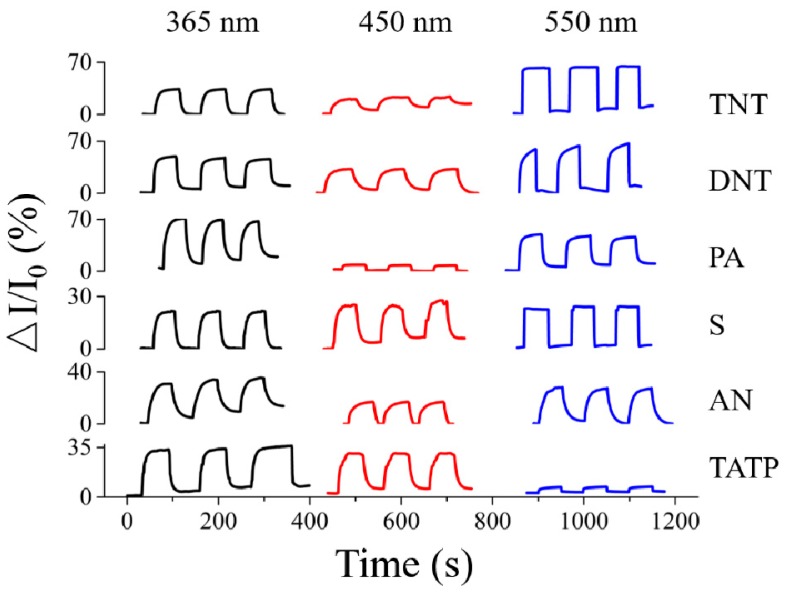
Response of TiO_2_/Aphen to different explosives under 365 nm, 450 nm and 550 nm illumination.

As mentioned above, Aphen functionalization is an effective strategy to enhance the light absorption range of TiO_2_. Thus, the sensor array can be constructed by only one TiO_2_/Aphen chip used under various illumination wavelengths. To demonstrate this concept, the sensing properties of the TiO_2_/Aphen to TNT, DNT, PA, S, AN and TATP were evaluated under 365 nm, 450 nm and 550 nm as depicted in Figure 6. The response (defined as (I_t_ − I_0_)/I_0_) of TiO_2_/Aphen to TNT, DNT, PA, S, AN and TATP vapor were 33.8%, 43.5%, 61.5%, 21.2%, 30.5% and 29% under 365 nm illumination, 6.5%, 27.4%, 8%, 21.1%, 17.1%, and 24.5% under 450 nm illumination, and 58.1%, 58.5%, 81.1%, 21.9%, 25.5% and 3.6% under 550 nm illumination, respectively.

Thus, the TiO_2_/Aphen shows distinctive responses to the tested explosives indicating that tailoring the excitation wavelength has an obvious effect on the sensing performance of TiO_2_/Aphen. For comparison, the sensing performance of the pure TiO_2_ to explosives’ vapor was characterized under the same conditions. As shown in Figure S2, the response of pure TiO_2_ to TNT, DNT, PA, S, AN and TATP vapor were 15%, 22.3%, 32.5%, 11.6%, 7.9% and 4% under 365 nm illumination. Interestingly, the pure TiO_2_ has almost no sensitivity to these explosives under illuminations of 450 nm and 550 nm.

**Figure 7:**
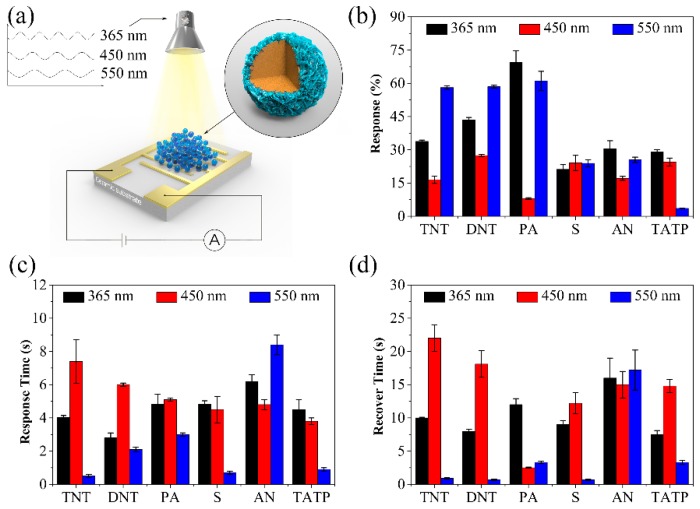
(**a**) Schematic diagram of sensor array realized by single TiO_2_/Aphen chip under various illumination wavelengths. Response (**b**), response time (**c**) and recover time (**d**) of TiO_2_/Aphen to different explosives under 365 nm, 450 nm and 550 nm illumination.

Therefore, by spinning coated TiO_2_/Aphen on the interdigitated electrode, a sensory array can be realized by tailoring the illumination wavelength used (Figure 7a). To gain a comprehensive understanding of the overall sensing performance of the sensory array, the sensing responses, response times and recovery times are statistically analyzed in Figure 7b–d. As shown in Figure 7b, the responses of the TiO_2_/Aphen to nitroaromatic explosives (TNT, DNT, PA) are 33.8%, 43.5%, and 69.5% under 365 nm illumination, and 58.1%, 58.5%, and 61.1% under 550 nm illumination, respectively. However, the responses of the TiO_2_/Aphen to improvised explosives (S, AN and TATP) are 21.2%, 30.5%, and 29% under 365 nm illumination, and 23.9%, 25.5%, and 36% under 550 nm illumination, respectively. Additionally, as depicted in Figure 6c, the response times of TiO_2_/Aphen to nitroaromatic explosives and improvised explosives are all within 10 s under the illuminations of 365 nm, 450 nm and 550 nm. The response time of TiO_2_/Aphen to TNT, DNT, PA, S, AN and TATP vapor were 4.04 s, 2.8 s, 4.83 s, 4.83 s, 6.2 s and 4.5 s under 365 nm illumination, 7.4 s, 6 s, 5.1 s, 4.5 s, 4.8 s, and 3.8 s under 450 nm illumination, and 0.5 s, 2.1 s, 3 s, 0.7 s, 8.4 s and 0.9 s under 550 nm illumination, respectively. TNT requires the longest recovery time (about 22 s) under 450 nm illumination, while DNT shows the shortest recover time (about 1 s) under 550 nm illumination. However, as shown in Figure S3 the response time and recover time of pure TiO_2_ to different explosives are more than 5 s under 365 nm illumination. 

**Figure 8:**
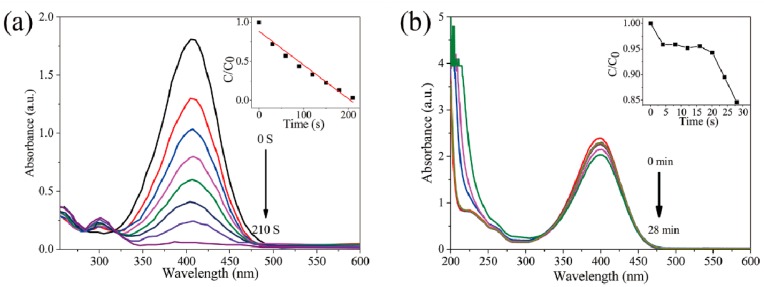
Successive UV-vis absorption spectra of the NaBH_4_ reduction of 4-nitrophenol catalyzed by TiO_2_/Aphen (**a**) and pure TiO_2_ (**b**).

The above experiments reveal that TiO_2_/Aphen has better sensing properties to nitroaromatic explosives than improvised explosives. This phenomenon may be attributed to the formation of a Meisenheimer complex between the electron-rich amino groups of Aphen and the electron-deficient aromatic rings of nitroaromatic explosives, coupled with strong charge transfer transitions. Thus, nitroaromatic explosives can anchor with TiO_2_ via Aphen bridge bonds and affected markedly the photocurrent of TiO_2_/Aphen. In addition, the photocatalytic mechanism may be the primary sensing mechanism after nitroaromatic explosives anchor to TiO_2_. To verify this hypothesis, the catalytic activity measurements of the TiO_2_/Aphen and pure TiO_2_ were based on the catalytic reduction of 4-nitrophenol aqueous solution as a substitute for TNT, DNT and PA vapors. As shown in Figure 8a, the absorption peak at 400 nm of 4-nitrophenol immediately decreased in presence of TiO_2_/Aphen under 550 nm illumination, owing to the photocatalytic reduction of 4-nitrophenolate to 4-aminophenol. The reduction reaction finished in 210 s with a reduction rate constant k = −0.004 s^−1^ which is determined from the plot of C/C_0_ *vs*. time. However, with addition of the pure TiO_2_, the absorption peak at 400 nm of 4-nitrophenol remains undiminished under 550 nm illumination for 28 min, attributed to the lake of visible light absorption of unmodified catalyst TiO_2_. Thus it can be seen that Aphen plays a key role in broadening the absorption band edge of TiO_2_ to the visible light region.

**Figure 9:**
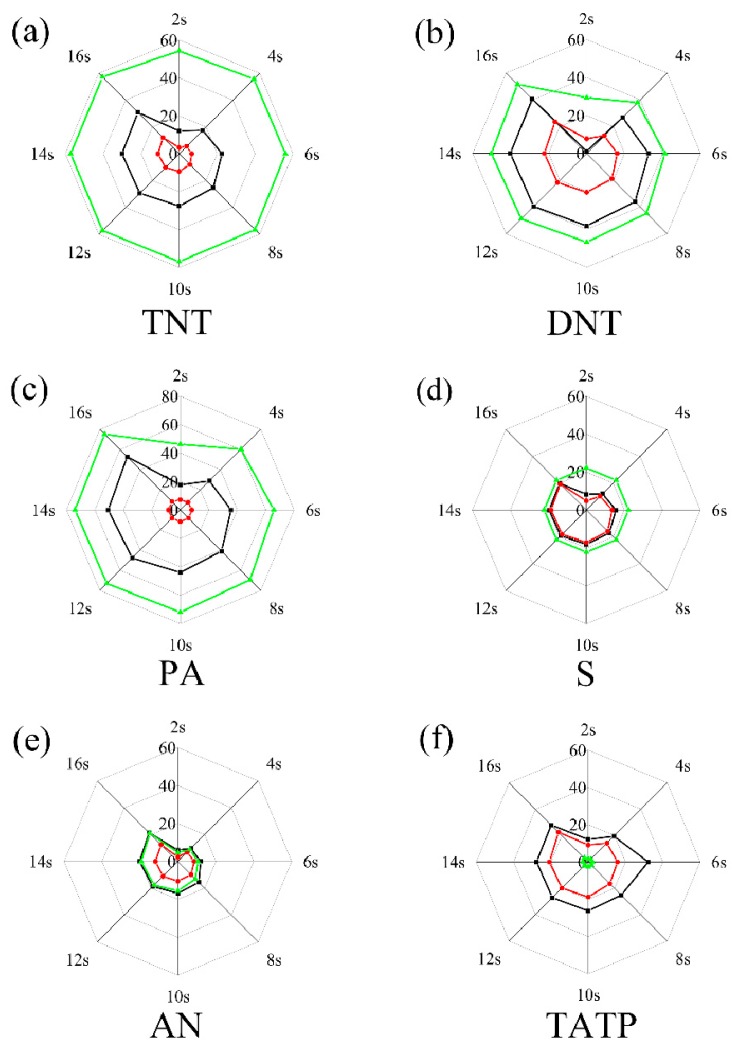
Fingerprint patterns collection of (**a**) TNT; (**b**) DNT; (**c**) PA; (**d**) S; (**e**) AN and (**f**) TATP by the response value at corresponding response time under 365 nm (black line), 450 nm (red line) and 550 nm (green line).

Generally, with a single sensor it is difficult to meet the actual needs of qualitative identification of different explosives. In other words, a single sensor can respond to different explosive vapors but cannot discriminate them from each other. Stated thus, the TiO_2_/Aphen shows a distinctive response, response time and recovery time to the tested explosives at different excitation wavelengths. Utilizing the cross-sensitivity and combining data analysis methods can imitate an artificial olfactory system and achieve the identification of different explosive vapors. Here, a sensor array based on the above single optoelectronic TiO_2_/Aphen sensor was constructed by regulating the excitation wavelength used. The response value at different response time was chosen as the eigenvalue. The fingerprint patterns were constructed by extracting every 1 s and 16 eigenvalues within 16 s in total (method A), or every 1 s and 8 eigenvalues within 8 s in total (method B) or every 2 s and 8 eigenvalues within 16 s in total (method C). As a result, the response to each kind of explosive at three wavelengths is different by method A and method C. However, method A process more data than method C. Thus, the response of the sensor is sampled every 2 seconds. Then these eigenvalues were grouped together to construct fingerprint patterns (Figure 9) of each explosive. It is found that each explosive has a unique fingerprint pattern, just like the loops and whorls on a human’s finger. The fingerprint pattern of TNT, for example, shows three whorls which are obtained from the response value at 2 s to 16 s under 365 nm (black line), 450 nm (red line) and 550 nm (green line). The whorls group of TNT is quite different from the that of DNT, PA and other improvised explosives, and so are those of DNT, PA, AN, S and TATP. Thus, one can identify explosives through their fingerprint patterns. Based on the fingerprint patterns, it promises to be a simple method to quickly identify these explosives from each other by machine identification.

The role of the moisture is also addressed in Figure S4. The response ratios of TNT are almost unchanged at 40% and 60 % of relative humidity (RH) and under the illumination of 365 nm. However, the response ratio of TNT decreased at 80% RH due to competitive adsorption of TNT and moisture on the surface of TiO_2_/Aphen. This result illustrates that the sensing response cannot be affected by ambient air below the 60% RH. These results have been added in the revised Supporting Information.

## 4. Conclusions

In conclusion, litchi-like TiO_2_ nanospheres with streaks on the surface were prepared by modifying the F ion content. Then Aphen-functionalized TiO_2_ nanospheres was successfully constructed by coordination interactions between Aphen and Ti ions on the surface of TiO_2_. Aphen plays a key role in broadening the absorption band edge of TiO_2_ into the visible light region. Thus a sensor array composed by only TiO_2_/Aphen sensors can be constructed by regulating the excitation wavelength in the visible region, which is significant for a low energy consumption, high selectivity and rapid response photoelectronic sensor. Moreover, a photocatalytic mechanism was proved the primary sensing mechanism of nitroaromatic explosives after nitroaromatic explosives anchor onto TiO_2_ by formation of a Meisenheimer complex. This work shines light on the realization of an artificial olfactory system to achieve the identification of different explosive vapors. The results demonstrate promising potential for low-cost practical applications as optoelectronic sensors for nitroaromatic explosive landmine detection and security checks. Undoubtedly, this approach also provide reference for the development of novel photocatalysts under visible light.

## Supplementary Materials

The following are available online at https://www.mdpi.com/1424-8220/19/10/2407/s1, Figure S1: SEM images of TiO_2_ regulated by (a) 0 mol, (b) 0.03 mol, (c) 0.06 mol, and (d) 0.12 mol of F ions. Figure S2: Response of pure TiO_2_ to TNT (a), DNT (b), PA (c), S (d), AN (e) and TATP (f) under 365 nm illumination. Figure S3: Response time and recover time of pure TiO_2_ to different explosives under 365 nm illumination. Figure S4: Effect of the humidity of TiO_2_/Aphen to TNT under the illumination of 365 nm.

## References

[1] Brown K.E., Greenfield M.T., McGrane S.D., Moore D.S. (2016). Advances in explosives analysis---part II: Photon and neutron methods. Anal. Bioanal. Chem..

[2] Whetstone Z.D., Kearfott K.J. (2014). A review of conventional explosives detection using active neutron interrogation. J. Radioanal. Nucl. Chem..

[3] Sun X., Wang Y., Lei Y. (2015). Fluorescence based explosive detection: from mechanisms to sensory materials. Chem. Soc. Rev..

[4] Zhang S.-R., Du D.-Y., Qin J.-S., Bao S.-J., Li S.-L., He W.-W., Lan Y.-Q., Shen P., Su Z.-M. (2014). A Fluorescent Sensor for Highly Selective Detection of Nitroaromatic Explosives Based on a 2D, Extremely Stable, Metal–Organic Framework. Chem. A Eur. J..

[5] Zhang L., Han Y., Zhu J., Zhai Y., Dong S. (2015). Simple and Sensitive Fluorescent and Electrochemical Trinitrotoluene Sensors Based on Aqueous Carbon Dots. Anal. Chem..

[6] Sisco E., Forbes T.P. (2015). Rapid detection of sugar alcohol precursors and corresponding nitrate ester explosives using direct analysis in real time mass spectrometry. Analyst.

[7] Mäkinen M., Nousiainen M., Sillanpää M. (2011). Ion spectrometric detection technologies for ultra-traces of explosives: A review. Mass Spectrom. Rev..

[8] Schramm S., Léonço D., Hubert C., Tabet J.-C., Bridoux M. (2015). Development and validation of an isotope dilution ultra-high performance liquid chromatography tandem mass spectrometry method for the reliable quantification of 1,3,5-Triamino-2,4,6-trinitrobenzene (TATB) and 14 other explosives and their degradation products in environmental water samples. Talanta.

[9] Schnorr J.M., van der Zwaag D., Walish J.J., Weizmann Y., Swager T.M. (2013). Sensory Arrays of Covalently Functionalized Single-Walled Carbon Nanotubes for Explosive Detection. Adv. Funct. Mater..

[10] Zheng Y., Xincun D., Shengli Z., Linjuan G., Baiyi Z., Zhaofeng W., Haibo Z. (2015). A High-Performance Nitro-Explosives Schottky Sensor Boosted by Interface Modulation. Adv. Funct. Mater..

[11] Qu J., Ge Y., Zu B., Li Y., Dou X. (2016). Transition-Metal-Doped p-Type ZnO Nanoparticle-Based Sensory Array for Instant Discrimination of Explosive Vapors. Small.

[12] Zhao J., Li N., Yu H., Wei Z., Liao M., Chen P., Wang S., Shi D., Sun Q., Zhang G. (2017). Highly Sensitive MoS2 Humidity Sensors Array for Noncontact Sensation. Adv. Mater..

[13] Lü X., Hao P., Xie G., Duan J., Gao L., Liu B. (2019). A Sensor Array Realized by a Single Flexible TiO_2_/POMs Film to Contactless Detection of Triacetone Triperoxide. Sensors.

[14] Wang S., Guan B.Y., Yu L., Lou X.W. (2017). (David) Rational Design of Three-Layered TiO_2_@Carbon@MoS_2_ Hierarchical Nanotubes for Enhanced Lithium Storage. Adv. Mater..

[15] Low J., Cheng B., Yu J. (2017). Surface modification and enhanced photocatalytic CO_2_ reduction performance of TiO_2_: A review. Appl. Surf. Sci..

[16] Chen X., Liu L., Huang F. (2015). Correction: Black titanium dioxide (TiO_2_) nanomaterials. Chem. Soc. Rev..

[17] Yan X., Xu Y., Tian B., Lei J., Zhang J., Wang L. (2018). Operando SERS self-monitoring photocatalytic oxidation of aminophenol on TiO_2_ semiconductor. Appl. Catal. B Environ..

[18] Yoshihara T., Murayama S., Masuda T., Kikuchi T., Yoshida K., Hosaka M., Tobita S. (2015). Mitochondria-targeted oxygen probes based on cationic iridium complexes with a 5-amino-1, 10-phenanthroline ligand. J. Photochem. Photobiol. A Chem..

[19] Shervedani R.K., Foroushani M.S., Dehaghi S.B. (2015). Functionalization of gold mercaptopropionic acid self-assembled monolayer with 5-amino-1,10-phenanthroline: Interaction with iron(II) and application for selective recognition of guanine. Electrochim. Acta.

[20] Feist B., Pilch M., Nycz J. (2019). Graphene oxide chemically modified with 5-amino-1,10-phenanthroline as sorbent for separation and preconcentration of trace amount of lead(II). Microchim. Acta.

[21] Smith G.F., Cagle F.W. (1947). The improved synthesis of 5-nitro-1, 10-phenanthroline. J. Org. Chem..

[22] Meng A., Zhang J., Xu D., Cheng B., Yu J. (2016). Enhanced photocatalytic H2-production activity of anatase TiO_2_ nanosheet by selectively depositing dual-cocatalysts on {101} and {001} facets. Appl. Catal. B Environ..

[23] Liu X., Dong G., Li S., Lu G., Bi Y. (2016). Direct Observation of Charge Separation on Anatase TiO_2_ Crystals with Selectively Etched {001} Facets. J. Am. Chem. Soc..

[24] Yuan Y.-J., Ye Z.-J., Lu H.-W., Hu B., Li Y.-H., Chen D.-Q., Zhong J.-S., Yu Z.-T., Zou Z.-G. (2016). Constructing Anatase TiO_2_ Nanosheets with Exposed (001) Facets/Layered MoS2 Two-Dimensional Nanojunctions for Enhanced Solar Hydrogen Generation. ACS Catal..

[25] Gnedenkov S.V., Opra D.P., Sinebryukhov S.L., Kuryavyi V.G., Ustinov А.Y., Sergienko V.I. (2015). Structural and electrochemical investigation of nanostructured C: TiO_2_–TiOF_2_ composite synthesized in plasma by an original method of pulsed high-voltage discharge. J. Alloys Compd..

[26] Zhao X., Wei G., Liu J., Wang Z., An C., Zhang J. (2016). Synthesis of heterostructured Pd@TiO_2_/TiOF_2_ nanohybrids with enhanced photocatalytic performance. Mater. Res. Bull..

[27] Jung M.-J., Kim Y., Lee Y.-S. (2017). Enhancement of the electrochemical capacitance of TiOF_2_ obtained via control of the crystal structure. J. Ind. Eng. Chem..

[28] Gao J., Lu C., Lu X. (2007). APhen-functionalized nanoparticles—Polymer fluorescent nanocomposites via ligand exchange and in situ bulk polymerization. J. Mater. Chem..

[29] Du Y., Lu C., Su Z., Gao J., Fu Y., Lu X. (2008). Synthesis and properties of transparent luminescent nanocomposites with surface functionalized semiconductor nanocrystals. J. Solid State Chem..

[30] Govindaiah P., Lee J.M., Jung Y.J., Lee S.J., Kim J.H. (2009). Tunable fluorescent poly (styrene-co-methacrylic acid)/Au-Aphen hybrid nanoparticles via surface immobilization. J. Mater. Chem.

[31] Isaacs M., Sykes A.G., Ronco S. (2006). Synthesis, characterization and photophysical properties of mixed ligand tris (polypyridyl) chromium (III) complexes, [Cr(phen)_2_L]^3+^. Inorganica Chim. Acta.

[32] Yang Y., Yin L.-C., Gong Y., Niu P., Wang J.-Q., Gu L., Chen X., Liu G., Wang L., Cheng H.-M. (2018). An Unusual Strong Visible-Light Absorption Band in Red Anatase TiO_2_ Photocatalyst Induced by Atomic Hydrogen-Occupied Oxygen Vacancies. Adv. Mater..

