# Supplementary material for: Fingerprinting of Nitroaromatic Explosives Realized by Aphen-functionalized Titanium Dioxide
Source: Sensors (Basel). 2019 May 27;19(10):2407. doi: 10.3390/s19102407 (PMC6566778; doi:10.3390/s19102407)
Supplement: Supplementary file 1 [file sensors-19-02407-s001.pdf]

Supporting Information

Fingerprinting of Nitroaromatic Explosives Realized by Aphen-functionalized Titanium Dioxide

Guanshun Xie and Bingxin Liu *

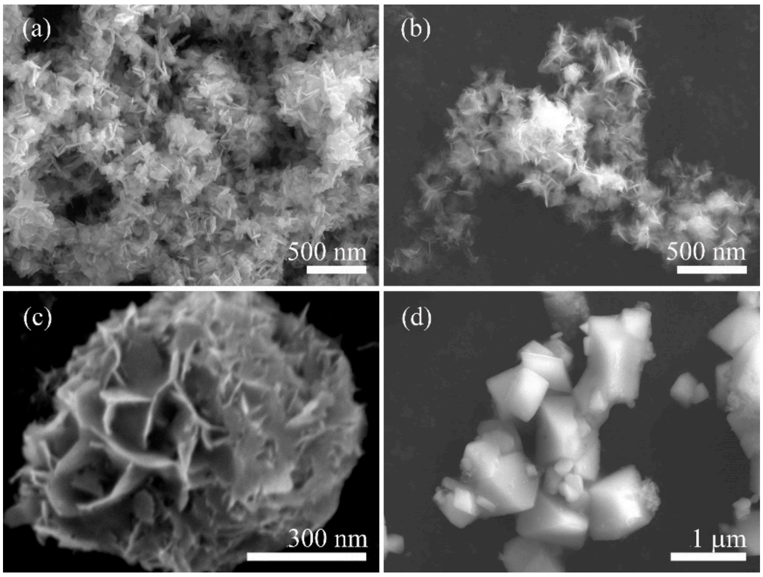

Figure S1. SEM images of TiO_2 regulated by (**a**) 0 mol, (**b**) 0.03 mol, (**c**) 0.06 mol, and (**d**) 0.12 mol of F ions.

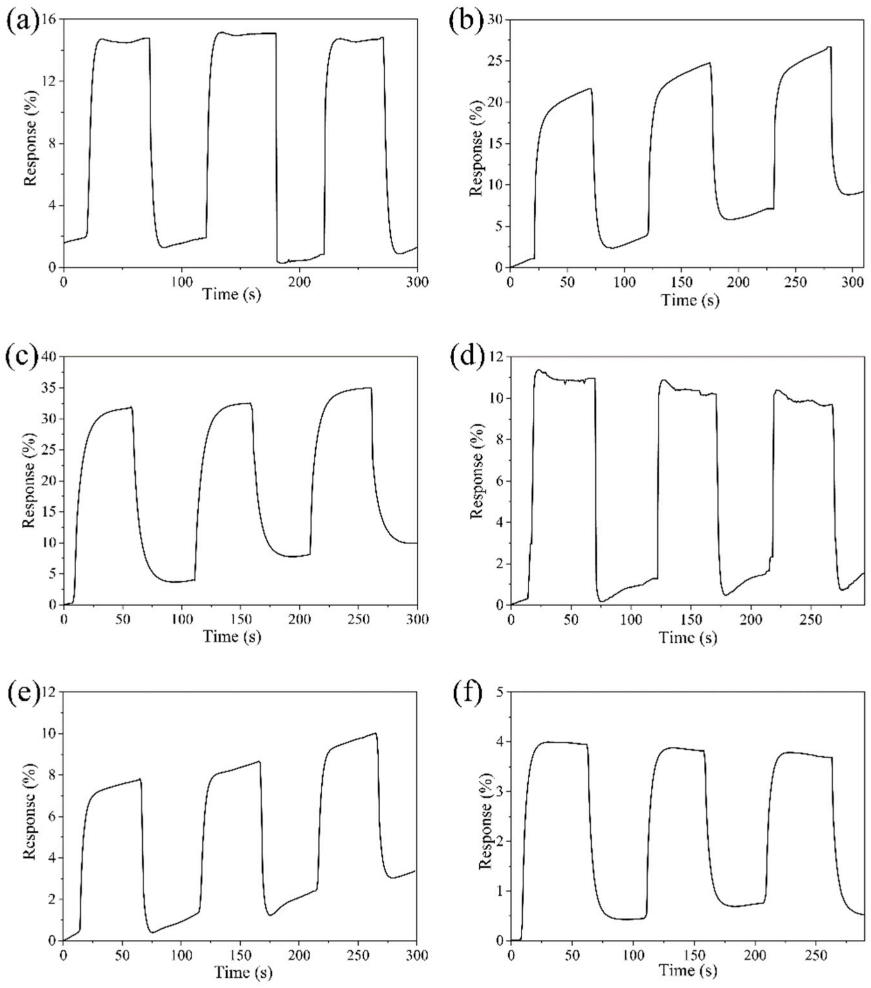

Figure S2. Response of pure TiO_2 to TNT (a), DNT (b), PA (c), S (d), AN (e) and TATP (f) under 365 nm illumination.

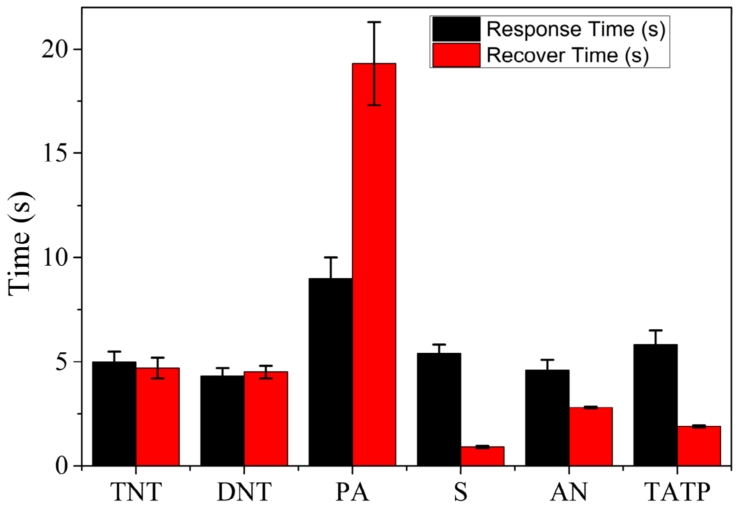

Figure S3. Response time and recover time of pure TiO_2 to different explosives under 365 nm illumination.

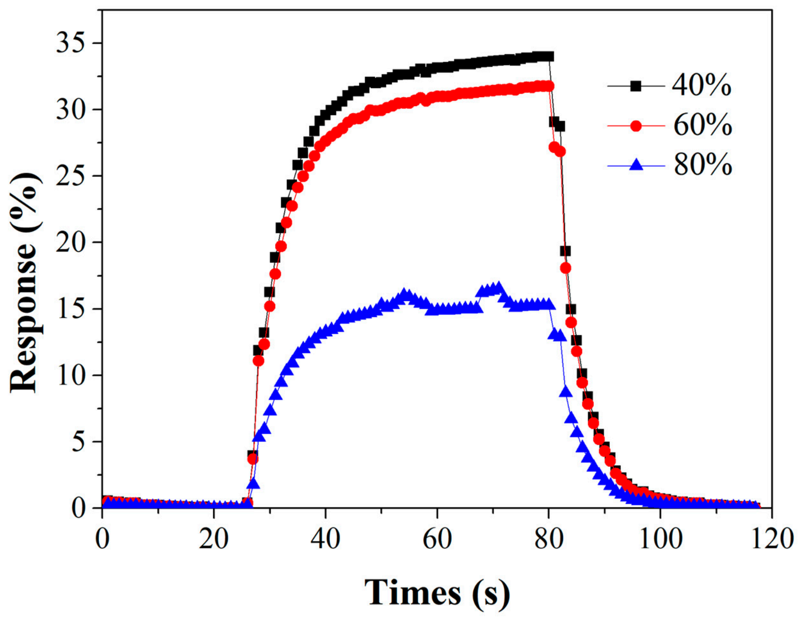

Figure S4. Effect of the humidity of TiO_2/Aphen to TNT under the illumination of 365 nm.
